# Trajectories of work disability among individuals with anxiety-, mood/affective-, or stress-related disorders in a primary healthcare setting

**DOI:** 10.1186/s12888-024-06068-5

**Published:** 2024-09-19

**Authors:** Magnus Helgesson, Emma Pettersson, Elin Lindsäter, Heidi Taipale, Antti Tanskanen, Ellenor Mittendorfer-Rutz, Alexis E. Cullen

**Affiliations:** 1 Department of Clinical Neuroscience, Division of Insurance Medicine, Karolinska Institutet, Stockholm, SE-17177 Sweden; 2 Department of Public Health and Caring Sciences, Public Health, Working Life and Rehabilitation, Uppsala University, Uppsala, SE-75122 Sweden; 3 Department of Clinical Neuroscience, Division of Psychology, Karolinska Institutet, Stockholm, SE-17177 Sweden; 4 Academic Primary Care Center, Region Stockholm, Sweden; 5 Department of Forensic Psychiatry, the University of Eastern Finland, Niuvanniemi Hospital, Kuopio, Finland; 6 School of Pharmacy, University of Eastern Finland, Kuopio, Finland; 7 Department of Psychosis Studies, Institute of Psychiatry, Psychology & Neuroscience, King’s College London, London, UK

**Keywords:** Sick leave, Psychiatric disorders, Depression, Anxiety disorder, Stress disorder primary care, Work capacity

## Abstract

**Background:**

Anxiety-, mood/affective-, or stress-related disorders affect up to one-third of individuals during their lives and often impact their ability to work. This study aimed to delineate trajectories of work disability (WD) among individuals diagnosed with anxiety-, mood/affective-, or stress-related disorder in primary healthcare and to examine associations between trajectory group membership and sociodemographic, clinical, and clinical-related factors.

**Methods:**

The study population included working-age individuals, aged 22–62 years, living in Stockholm County, Sweden, who experienced a new episode of any anxiety-, mood/affective, or stress-related disorder in primary healthcare in 2017 (*N* = 11,304). Data were obtained from Swedish national and regional registers and were linked using pseudonymised unique personal identification numbers. The primary outcome was days with WD (sum of sickness absence and disability pension days) during the three years before and three years after a diagnosis of anxiety-, mood/affective-, or stress-related disorders in primary healthcare. A zero-inflated Poisson group-based trajectory model was used to identify groups of individuals with similar patterns of WD over the study period, with a multinomial logistic regression used to examine associations of sociodemographic, clinical, and clinical-related factors with trajectory group membership.

**Results:**

Four distinct trajectory groups were found, *high increasing* (5.1%), with high levels, from 16 to 80 days of WD in six-monthly intervals during follow-up, *peak* (11.1%), with a peak in WD, up to 32 days of WD, around the time of the diagnosis, *low increasing* (12.8%), with an increase in days of WD from 4 to 22 during the study period, and *constant low* (71.1%), with almost no WD over the study period. In multinomial regression models, diagnostic category, psychotropic medication use, a diagnosis of a psychiatric disorder within secondary healthcare, age at diagnosis, and occupation were associated with WD trajectory groups.

**Conclusions:**

Around two-thirds of individuals treated for a new episode of any anxiety-, mood/affective-, or stress-related disorder in primary healthcare have an excellent prognosis regarding WD. Several sociodemographic and clinical characteristics were associated with group membership; these factors could identify individuals at risk of long-term welfare dependency and who might benefit from interventions to promote a return to work.

**Supplementary Information:**

The online version contains supplementary material available at 10.1186/s12888-024-06068-5.

## Background

Anxiety-, mood/affective-, or stress-related disorders affect around one-third of individuals during their working lives [1]. These disorders often affect the ability to work and are among the most common causes of long-term sickness absence and the granting of disability pension in Sweden [2–5]. These disorders, particularly stress-related disorders, have increased substantially during the last two decades [4, 6]. Possible reasons for this might be that work demands have increased simultaneously, and help-seeking for these disorders has changed [7]. Inability to work because of anxiety-, mood/affective-, or stress-related disorders can be very costly, both from a social and an employer perspective, but not least for the individuals who risk permanent exclusion from the labour market and risk being dependent on welfare benefits. A previous population-based study reported that as many as two-thirds of young adults (aged 20 to 35 years) treated for their first episode of a common mental disorder (CMD, which included anxiety, mood/affective, or stress-related disorders) in specialised healthcare settings had poor prognoses concerning later labour market participation [5]. This may be expected, as those treated for CMDs in secondary healthcare can be expected to have more severe clinical presentations than those treated within primary care and, therefore, are more likely to have long periods outside the labour market. However, given that most individuals who develop anxiety-, mood/affective-, or stress-related disorders are treated solely within primary care settings (without referral to secondary care), it is crucial to determine the long-term work trajectories for people treated for anxiety-, mood/affective-, or stress-related disorders in this setting as this represents the vast majority of people with these disorders [8].

Identifying risk and protective factors for long-term labour market marginalisation among individuals with anxiety-, mood/affective-, or stress-related disorders is essential to developing strategies to promote recovery. Systematic psychological therapies (e.g., cognitive behavioural therapy and psychodynamic psychotherapy) and pharmacological therapies (e.g., antidepressants) have been previously found to be associated with some improvements in symptoms and functioning among people with CMDs [9–13]. However, previous studies investigating associations between treatment factors and work disability among individuals with CMD have typically utilised small samples, making it difficult to detect differences between different subtypes of CMDs [14].

To address these knowledge gaps, we used data from national and regional registers obtained from Swedish healthcare and other government authorities to examine trajectories of work disability among individuals with anxiety-, mood/affective-, or stress-related disorders. These high-quality registers capture periods of work disability and provide information on sociodemographic and work-related factors (e.g., gender, age, educational level and profession) as well as clinical-related factors (e.g., type of disorder, comorbid somatic and psychiatric conditions and psychological and pharmacological therapies) that may be associated with trajectories of work disability [2, 5].

Days out of work due to illness is a significant public health issue as more extended periods of work disability tend to increase labour market marginalisation as well as declining mental illness. Finding individuals at most risk of ending up outside the labour market is a prioritised issue to avoid permanent exclusion from the labour market and worsening of mental health [15, 16]. We, therefore, aimed to delineate trajectories of work disability among individuals diagnosed with a new episode of any anxiety-, mood/affective-, or stress-related disorder in primary healthcare. Our primary aim was to assess the heterogeneity in the population and determine how belonging to these trajectory groups was associated with sociodemographic, work-related, and clinical factors.

## Methods

### Setting

In Sweden, primary healthcare has been commissioned by the government to prevent and treat individuals with mild to moderate mental health problems, including anxiety-, mood/affective-, or stress-related disorders. Patients with severe psychiatric disorders or elevated risk for suicide are immediately referred to secondary psychiatric clinics (to which they may also self-refer). However, most patients are referred from primary to secondary healthcare only after undergoing one or several treatment attempts in the primary care setting.

### Data sources

Data were sourced from various Swedish national and regional registers, linked individually using pseudonymised unique personal identification numbers. The VAL database is the data storage solution for healthcare visits within Region Stockholm, Sweden, which includes individual-level data on primary care visits in Stockholm County. The Longitudinal Integration Database for Health Insurance and Labour Market Studies (LISA) contains data on sociodemographic and work-related factors. The Micro-Data for Analyses of Social Insurance (MiDAS) included data on work disability. The National Patient Register (NPR) included data on treatment in inpatient and specialised outpatient healthcare. The Prescribed Drug Register (PDR) included information on drug purchases. An overview of the registers utilised is provided in Supplementary Table 1.

### Study population

In the VAL database, we first identified all individuals with any recorded diagnosis of anxiety-, mood/affective-, or stress-related disorders during 2017 (*N* = 124,963) [17]. The study population included working-aged individuals in Stockholm experiencing a new episode of anxiety- (International Statistical Classifications of Diseases – version 10 (ICD-10) [18]: F40-F42), mood/affective- (ICD-10: F32-F39) or stress-related disorders (ICD-10: F40-F42) in primary healthcare during the year 2017. This year was chosen to have data for healthcare visits three years before and after the inclusion date, as reliable diagnostic data has only been available since 2014. Individuals were required to be registered residents in Stockholm County and of working age (22–62 years) during each calendar year between 2014 and 2019, inclusive (*N* = 84,275). The reason for choosing 22 years as the lower age range was to ensure that all participants in the study were eligible to be granted disability pension three years before inclusion, as it is not possible to receive disability pension before the age of 19 in Sweden. To identify individuals experiencing a new episode of anxiety-, mood/affective-, or stress-related disorder, that is, a visit to primary healthcare due to any of these disorders in 2017, without having a visit to either primary or secondary healthcare due to anxiety-, mood/affective-, or stress-related disorders three years before, all individuals with an inpatient, specialised outpatient, or primary care contact for any anxiety-, mood/affective-, or stress-related disorders diagnosis in the three years before cohort entry were excluded from the study (*N* = 32,868). Further exclusions were made for individuals who, at any point in the three years before cohort entry, had spells of sickness absence attributed to any anxiety-, mood/affective-, or stress-related disorder, purchased psychotropic medications indicated for anxiety-, mood/affective-, or stress-related disorders (Anatomical Therapeutic Chemical Classification, ATC: N06A, N05B, N05C, R06AD01) or had visits in primary care where psychological therapy (including both systematic and non-systematic psychological treatment) had been administered (*N* = 11,635). Finally, individuals with severe mental illnesses, including schizophrenia-spectrum disorders (ICD-10: F20-F29), bipolar disorder (ICD-10: F30-F31), and organic mental disorders (ICD-10: F00-F09), and those who had been granted full-time disability pension before cohort entry, were excluded as these conditions are associated with high levels of disability and therefore may confound the association between anxiety-, mood/affective-, and stress-related disorders and our primary outcome. As anxiety-, mood/affective-, and stress-related disorders are highly co-morbid with many different psychological disorders, we accounted for other mental comorbidities (ICD-10: F10-F19 and F44-F99) by including these as covariates in the analyses. The final study population consisted of 11,304 individuals.

### Outcome measures

The primary outcome of this study was days of work disability, defined as the sum of net days, that is, one combined day of work disability. Here, one day, two half days or four quarters of a day equals one net day with sickness absence and disability pension benefits. Work disability was measured at six-monthly intervals over the study period, which spanned three years before and three years after cohort entry (i.e., the date of the first diagnosis of a mood/affective-, anxiety-, or stress-related disorder in 2017).

All individuals aged 19–64 in Sweden can be granted disability pension if their work capacity is reduced due to illness. Individuals aged 19–29 may be granted time-restricted disability pension if they have an impaired work capacity or have not completed compulsory education. Individuals aged 30–64 years may be granted permanent disability pension if they have an expected lifelong duration of reduced work capacity. Individuals aged 16 and above with a certain income level from work can receive benefits concerning sickness absence. Employers are typically responsible for payment of sickness absence benefits during the first 14 days of a sick leave spell, after which the Social Insurance Agency covers payments. For this reason, only information on sickness absence spells longer than 14 days is readily available in national registers and was used to compute the outcome measure.

When computing the outcome measure, the extent to which sickness absence and disability pension were granted were taken into account; for example, two days of part-time sickness absence at a 50% extent was combined to equal one net day of work disability. In the study, disability pension and sickness absence were summed up to encompass days on work disability.

### Sociodemographic and work-related factors

Sociodemographic factors included gender (male vs. female), country of birth (Sweden vs. elsewhere), years of education (0–9 vs. 10–12 vs. > 12 years), family composition (cohabiting with no children living at home vs. cohabiting with children living at home vs. single with no children living at home vs. single with children living at home), place of residence (large cities vs. other), and days of unemployment (none vs. any). These factors were measured on the 31st of December 2016, the year before the diagnosis of an anxiety-, mood/affective-, or stress-related disorder. The occupation group was measured in November 2016 based on employment status. It was categorised according to the Swedish Standard Classification of Occupations 2012 (SSYK 2012) as (1) occupations with university requirements (SSYK 2012): 1111–3522), (2) administrative roles and customer service, (SSYK 2012: 4111–5419), (3) non-managerial workers (SSYK 2012: 6111–9629, 0110–0310), (4) not gainfully employed and (5) no information on the occupation. Age was estimated during the cohort entry year (2017) and categorised as 22–29 vs. 30–39 vs. 40–49 vs. 50–59 vs. 60–62 years.

.

### Clinically related factors

Type of diagnostic category included anxiety disorders (ICD-10: F40-F42), mood/affective disorders (ICD-10: F32-F39), and stress-related disorders (ICD-10: F43), that is, reaction to severe stress, adjustment disorders, acute stress and post-traumatic stress disorders (PTSD) from primary healthcare. The treatment variables included the receipt of systematic psychological treatment (defined as psychodynamic treatment (PDT), cognitive psychological therapy (CT), cognitive behavioural therapy (CBT), mentalization-based therapy (MBT), Eye Movement Desensitization and Reprocessing (EMDR), systemic therapy (ST), dialectical-behavioural therapy (DBT), interpersonal therapy (IPT), and other psychological treatment), administered by psychotherapists in primary care (treatment codes (KVÅ codes) in the registers: DU008- DU011, DU013-DU014, DU020-DU022) and purchases of psychotropic medications including antidepressants (Anatomical Therapeutic Chemical code (ATC): N06A), anxiolytics (ATC: N05B), hypnotics and sedatives (ATC: N05C) and alimemazin (R06AD01). These exposures were measured over the three years following the new episode of any anxiety-, mood/affective-, or stress-related disorder. Moreover, diagnoses of visits from inpatient/specialised outpatient healthcare were measured for all psychiatric disorders (ICD-10: F00-F99).

### Statistical methods

Zero-inflated Poisson group-based trajectory models (ZIP GBTM) were used to identify groups of individuals with similar patterns of work disability over the study period [19]. This method is a type of finite mixture model appropriate for analysing evolving longitudinal processes. Instead of assuming the existence and forms of hypothesised trajectories of work disability, the method can identify distinct and meaningful patterns of work disability from the data itself [20]. These models assume the presence of unobserved latent groups, where each group follows a unique trajectory of work disability over time. GBTMs can accommodate various data types; in this instance, a zero-inflated Poisson (ZIP) distribution was determined to provide the best fit based on the distribution of work disability days (this outcome is highly skewed, with a large proportion of individuals having zero days of work disability). A ZIP model provides a more appropriate fit for data with these characteristics than other models such as the standard Poisson or a censored normal model). Proportions of the population belonging to each latent group and the probability of group membership can also be estimated from the model.

Due to the explorative nature of the GBTM method, different models with varying numbers of latent groups and other sets of parameter constraints were compared to identify the best-fitting model. Nagin [20] suggests a practical two-stage approach for model selection. The first stage involves determining the optimal number of trajectory groups, and the second stage involves identifying the optimal polynomial form of the trajectories. We fitted models that included two to six trajectory groups with varying polynomial shapes (from linear to quartic). Several diagnostic metrics were considered when selecting the optimal model; these included the Akaike information criterion (AIC), the Bayesian information criterion (BIC), the average posterior probability of assignment (APPA), the mean squared error (MSE), relative entropy, and the and the odds of correct classification (OCC).

After identifying the optimal trajectories of work disability, individuals were assigned trajectory groups based on estimated group membership probabilities. A multinomial logistic regression was then conducted to examine the associations between sociodemographic, work-related and clinical covariates with trajectory group assignment. Although we refer to these as exposures, they are measured at a single time point. Still, the outcome measure incorporates data regarding work disability from before and after diagnosis. Each covariate was excluded from the saturated model to assess the relative contribution of the different covariates; the reduced and saturated models were then compared using likelihood-based metrics, including likelihood ratio tests and Nagelkerke R^2^. Marginal effect plots are presented for the exposures and the covariates with the highest relative importance in the models. Statistical analyses were performed in R (version 4.2.2) using the ‘latrend’, ‘nnet’ and ‘effects’ packages.

## Results

### Characteristics

The characteristics of the 11,304 individuals treated for a new episode of any anxiety-, mood/affective-, or stress-related disorder in primary care during 2017 are presented in Table 1. Most individuals (53.8%) were aged 30 to 49 years at cohort entry and were born in Sweden (72.7%); just over half (50.1%) were female. The most common family situation category was single without children (47.1%), and nearly half (49.0%) had more than twelve years of education. With regards to work-related factors, less than 1 in 10 (8.3%) were unemployed in the year before cohort entry; the most common occupations were those that required a university education (40.8%), followed by administration and customer service occupations (25.2%).

**Table 1:** Baseline characteristics, overall and across trajectory groups for different trajectory groups for individuals diagnosed with CMDs in Stockholm County in 2017 (*n* = 11,304)

	Overall	High increasing	Peak	Low increasing	Constant low
	(*N* = 11,304)	(*N* = 567)	(*N* = 1,155)	(*N* = 1,361)	(*N* = 8,221)
**Sociodemographic and work-related factors**					
**Age**					
22–29	2075 (18.4%)	66 (11.6%)	136 (11.8%)	190 (14.0%)	1683 (20.5%)
30–39	2986 (26.4%)	122 (21.5%)	313 (27.1%)	334 (24.5%)	2217 (27.0%)
40–49	3101 (27.4%)	155 (27.3%)	334 (28.9%)	357 (26.2%)	2255 (27.4%)
50–59	2603 (23.0%)	168 (29.6%)	299 (25.9%)	389 (28.6%)	1747 (21.3%)
60–62	539 (4.8%)	56 (9.9%)	73 (6.3%)	91 (6.7%)	319 (3.9%)
**Sex**					
Female	5660 (50.1%)	303 (53.4%)	679 (58.8%)	788 (57.9%)	3890 (47.3%)
Male	5644 (49.9%)	264 (46.6%)	476 (41.2%)	573 (42.1%)	4331 (52.7%)
**Family composition**					
Cohabitant, no children	1432 (12.7%)	103 (18.2%)	153 (13.2%)	218 (16.0%)	958 (11.7%)
Cohabitant, with children	3821 (33.8%)	157 (27.7%)	419 (36.3%)	462 (33.9%)	2783 (33.9%)
Single, no children	5320 (47.1%)	261 (46.0%)	487 (42.2%)	579 (42.5%)	3993 (48.6%)
Single, with children	731 (6.5%)	46 (8.1%)	96 (8.3%)	102 (7.5%)	487 (5.9%)
**Country of birth**					
Sweden	8221 (72.7%)	383 (67.5%)	827 (71.6%)	976 (71.7%)	6035 (73.4%)
Elsewhere	3083 (27.3%)	184 (32.5%)	328 (28.4%)	385 (28.3%)	2186 (26.6%)
**Place of residence**					
Big cities	10,998 (97.3%)	548 (96.6%)	1108 (95.9%)	1313 (96.5%)	8029 (97.7%)
Intermediate/small cities	306 (2.7%)	19 (3.4%)	47 (4.1%)	48 (3.5%)	192 (2.3%)
**Year of education**					
0–9 years	1256 (11.1%)	98 (17.3%)	133 (11.5%)	159 (11.7%)	866 (10.5%)
10–12 years	4508 (39.9%)	268 (47.3%)	495 (42.9%)	598 (43.9%)	3147 (38.3%)
> 12 years	5540 (49.0%)	201 (35.4%)	527 (45.6%)	604 (44.4%)	4208 (51.2%)
**Unemployment at baseline**					
none	10,365 (91.7%)	499 (88.0%)	1084 (93.9%)	1260 (92.6%)	7522 (91.5%)
any	939 (8.3%)	68 (12.0%)	71 (6.1%)	101 (7.4%)	699 (8.5%)
**Profession**					
Occupations with university requirements^1^	4607 (40.8%)	166 (29.3%)	515 (44.6%)	528 (38.8%)	3398 (41.3%)
Administration and customer service^2^	2854 (25.2%)	160 (28.2%)	336 (29.1%)	427 (31.4%)	1931 (23.5%)
Non-managerial workers^3^	1339 (11.8%)	85 (15.0%)	131 (11.3%)	199 (14.6%)	924 (11.2%)
Not gainfully employed	1275 (11.3%)	86 (15.2%)	57 (4.9%)	72 (5.3%)	1060 (12.9%)
No information on the occupation	1229 (10.9%)	70 (12.3%)	116 (10.0%)	135 (9.9%)	908 (11.0%)
**Clinical-related factors**					
**Type of diagnostic category**					
Anxiety disorders (ICD10: F40-F42)	3105 (27.5%)	89 (15.7%)	148 (12.8%)	227 (16.7%)	2641 (32.1%)
Mood/affective disorders (ICD10: F32-F39)	1619 (14.3%)	74 (13.1%)	111 (9.6%)	147 (10.8%)	1287 (15.7%)
Stress-related disorders (ICD10: F43)	3749 (33.2%)	156 (27.5%)	425 (36.8%)	474 (34.8%)	2694 (32.8%)
Anxiety- and mood/affective disorders	685 (6.1%)	47 (8.3%)	47 (4.1%)	89 (6.5%)	502 (6.1%)
Anxiety- and stress-related disorders	1107 (9.8%)	73 (12.9%)	178 (15.4%)	213 (15.7%)	643 (7.8%)
Mood/affective and stress-related disorders	639 (5.7%)	72 (12.7%)	147 (12.7%)	121 (8.9%)	299 (3.6%)
Anxiety- mood/affective- and stress-related disorders,	400 (3.5%)	56 (9.9%)	99 (8.6%)	90 (6.6%)	155 (1.9%)
**Comorbid psychological disorders in primary care**	452 (4.0%)	48 (8.5%)	34 (2.9%)	63 (4.6%)	307 (3.7%)
**(Prior** ^**4**^ **) Psychiatric diagnosis** ^**5**^ **from inpatient/specialised outpatient healthcare**	296 (2.6%)	19 (3.4%)	21 (1.8%)	32 (2.4%)	224 (2.7%)
**(Post** ^**6**^ **) Psychiatric diagnosis** ^**5**^ **from inpatient/specialised outpatient healthcare**	2008 (17.8%)	300 (52.9%)	376 (32.6%)	322 (23.7%)	1010 (12.3%)
**(Prior** ^**4**^ **) Somatic diagnosis** ^**6**^ **from inpatient/specialised outpatient healthcare**	7282 (64.4%)	446 (78.7%)	795 (68.8%)	1021 (75.0%)	5020 (61.1%)
**(Post** ^**6**^ **) Somatic diagnosis** ^**6**^ **from inpatient/specialised outpatient healthcare**	7102 (62.8%)	443 (78.1%)	799 (69.2%)	1015 (74.6%)	4845 (58.9%)
**Any systematic psychological treatment therapy** ^**8**^					
No psychological therapy	7884 (69.7%)	341 (60.1%)	778 (67.4%)	905 (66.5%)	5860 (71.3%)
In year one	2076 (18.4%)	119 (21.0%)	200 (17.3%)	236 (17.3%)	1521 (18.5%)
In years two to three (only)	745 (6.6%)	50 (8.8%)	93 (8.1%)	130 (9.6%)	472 (5.7%)
In years one and two to three	599 (5.3%)	57 (10.1%)	84 (7.3%)	90 (6.6%)	368 (4.5%)
**Purchase of psychotropics** ^**9**^					
No psychotropic purchases	5060 (44.8%)	153 (27.0%)	386 (33.4%)	519 (38.1%)	4002 (48.7%)
In year one	2740 (24.2%)	104 (18.3%)	317 (27.4%)	324 (23.8%)	1995 (24.3%)
In years two to three (only)	717 (6.3%)	47 (8.3%)	83 (7.2%)	125 (9.2%)	462 (5.6%)
In years one and two to three	2787 (24.7%)	263 (46.4%)	369 (31.9%)	393 (28.9%)	1762 (21.4%)
**Purchase of any other psychotropics** ^**10**^	553 (4.9%)	89 (15.7%)	65 (5.6%)	80 (5.9%)	319 (3.9%)

^1^ Swedish Standard Classifications of Occupations 2012 (SSYK 2012): 1111–3522, for example managers, physicians, nurses and teachers

^2^ SSYK 2012: 4111–5419, for example, economic assistants, store workers, postmen

^3^ SSYK 2012: 6111–9629, 0110–0310, for example, construction workers, military personnel, farmers

^4^ Measured in the three years before cohort entry

^5^ International Statistical Classifications of Diseases – version 10 (ICD10): F00-F99

^6^ Measured in the three years post to cohort entry

^7^ All ICD10 codes A-Z excluding F, O, P, Q, R, U, Z

^8^ Systematic psychological treatment included psychodynamic treatment (PDT), cognitive psychological therapy (CT), cognitive behavioural therapy (CBT), mentalization-based therapy (MBT), Eye Movement Desensitization and Reprocessing (EMDR), systemic therapy (ST), dialectical-behavioural therapy (DBT), interpersonal therapy (IPT), and other psychological treatment (KVÅ codes: DU008- DU011, DU013-DU014, DU020-DU022)

^9^ Anatomical Therapeutic Chemical Classification (ATC): N06A, N05B, N05C, R06AD01

^10^ ATC: N05A, N03AF01, N03AG01, N03AX09, N05AN01, N06B, N07BB, N07BC, N06CA

About 3 out of 10 individuals received at least one session of systematic psychological treatment during the three years after the diagnosis of any anxiety-, mood/affective-, or stress-related disorder, whereas the majority had their session of systematic psychological treatment within one year after the diagnosis. At least half of the population purchased psychotropic medications during the three-year follow-up (55.2%). In the study, 75% of individuals were diagnosed with just one of the three categories: anxiety-, mood/affective-, or stress-related disorders, while the remainder (25%) had combinations of these conditions. Over half of the study population (52.2%) had a diagnosis of a stress-related disorder. Within this group, 33.2% were diagnosed solely with a stress-related disorder, and 19.0% had a stress-related disorder alongside an anxiety- or mood/affective disorder. Anxiety disorders were found in just under half (46.9%) of the population, where 27.5% experienced solely an anxiety disorder and 19.4% had an anxiety disorder combined with a mood/affective- or stress-related disorder. Mood/affective disorders were found in about one-third (29.6%) of the population, with 14.3% having only a mood/affective disorder and 15.3% experiencing this alongside an anxiety- or stress-related disorder. The most common combination of disorders found in the study was between stress-related and anxiety disorders, which affected 9.8% of the population.

### Trajectory groups

**Fig. 1:**
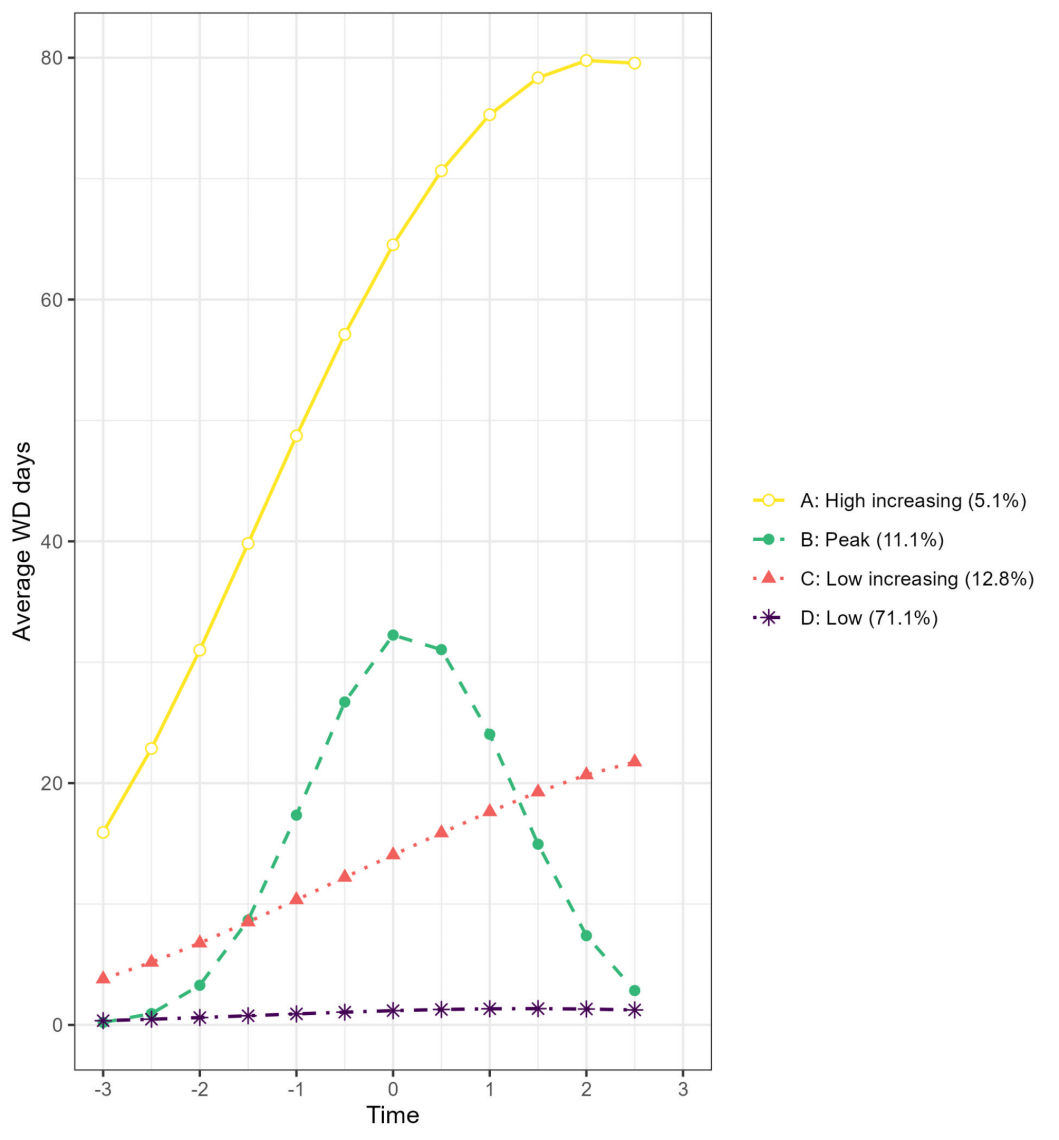
Trajectory groups of work disability around the year 2017 (t0) from 2014 (-3) to 2020 [3] divided into six-month intervals

The model with four trajectory groups produced the lowest AIC, BIC and MSE metrics, while the remaining metrics were similar across all models. When identifying the optimal polynomial form of the trajectories, AIC and BIC decreased marginally as complexity was added to the model; however, the four-group quadratic model was selected as the most parsimonious model as it produced the highest APPA and relative entropy metrics and with the lowest MSE. The average levels of work disability across the study period, estimated from the overall model, are shown in Fig. 1 for the four WD trajectory groups. The trajectory groups were: *high increasing* (with 5.1% of the included population), characterised by a high starting level of average work disability (16 *six-monthly intervals* ), which continued to increase during follow-up; *peak* (11.1%), characterised by lower levels of average work disability at the start and end of the study period but with a peak around the time of diagnosis of the new episode of anxiety-, mood/affective-, or stress-related disorders; *low increasing* (12.8%), characterised by a lower starting level of average work disability which increased over the entire study period; and c*onstant low* (71.1%), with low levels of average work disability during the whole study period.

### Characteristics and predictors across trajectory groups

**Fig. 2:**
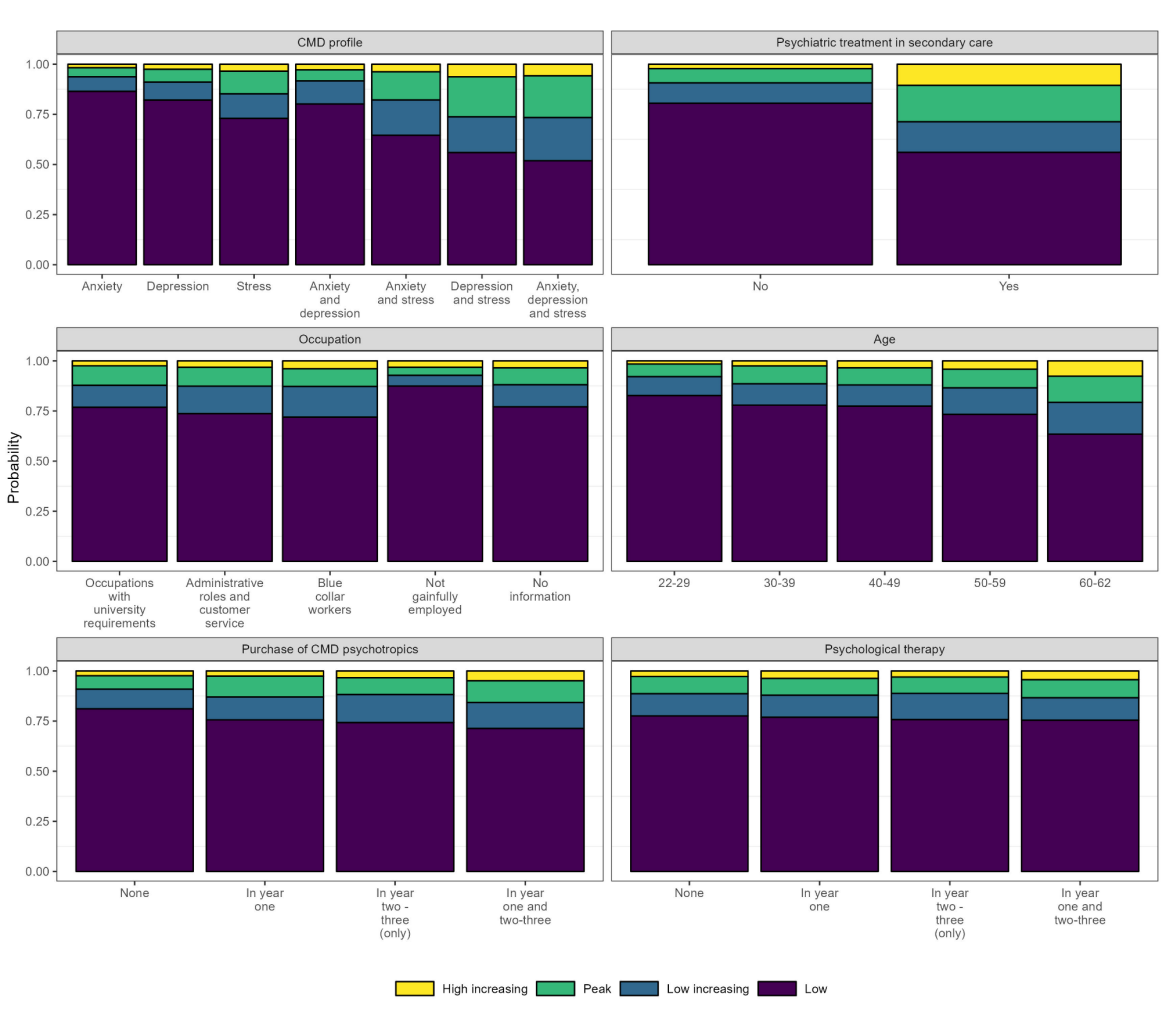
Predicted probability plots showing the probability of trajectory group membership

In the multinomial regression model, the most important predictors of trajectory group membership were found to be the type of diagnostic category, i.e., anxiety disorders, mood/affective disorders, and stress-related disorders (4.8% difference in R^2^ when introduced into the model), diagnosis of any psychiatric disorder in secondary healthcare (4.0%) following the new episode of anxiety-, mood/affective-, or stress-related disorders, profession (1.2%), age at cohort entry (1.0%) and purchases of psychotropic medication (1.0%). Marginal effect plots, showing the estimated probability of trajectory group membership across different levels of these covariates, along with psychological treatment, are shown in Fig. 2.

The fully adjusted multinomial logistic regression with estimated odds ratios (OR) and 95% confidence intervals (CI) are shown in Table 2, where the *high-increasing*, *peak* and *low-increasing* trajectory groups were compared with the *constant low* group which consisted of a higher proportion of 22–29-year-olds (20.5%) and fewer individuals aged over 50 (25.2%) compared to the other classes with higher levels of work disability (11.6 − 14.0% 22–29 years old; 32.2 − 39.5% over 50, Table 1). The *Constant low* group also comprised a high proportion of individuals with a high educational level (51.2%).

**Table 2:** Fully adjusted odds ratios (ORs) of trajectory group membership associated with sociodemographic, work- and health-related factors, compared to the reference group (*constant low* trajectory of work disability) in individuals with anxiety-, mood/affective-, or stress-related disorders treated in primary health care settings in Stockholm, Sweden

Characteristic	High increasing vs. Constant low	Peak vs. Constant low	Low increasing vs. Constant low	Likelihood-ratio test	Pseudo R2 difference^1^
	OR (95% CI)	OR (95% CI)	OR (95% CI)	*p*-value	%
**Sociodemographic and work-related factors** ^**2**^					
***Age***				**< 0.001**	1.038
22–29	Ref	Ref	Ref		
30–39	**1.68 (1.20–2.35)**	**1.50 (1.18–1.89)**	1.21 (0.98–1.50)		
40–49	**2.36 (1.67–3.35)**	**1.44 (1.12–1.84)**	1.20 (0.96–1.50)		
50–59	**2.94 (2.10–4.13)**	**1.66 (1.30–2.12)**	**1.59 (1.29–1.97)**		
60–62	**6.28 (4.08–9.68)**	**2.68 (1.90–3.78)**	**2.21 (1.63–3.01)**		
***Sex***				**< 0.001**	0.156
Female	Ref	Ref	Ref		
Male	0.91 (0.74–1.11)	**0.77 (0.66–0.89)**	**0.82 (0.72–0.94)**		
***Family composition***					
Cohabitant, no children	Ref	Ref	Ref	0.482	0.075
Cohabitant with children	0.76 (0.56–1.03)	1.12 (0.89–1.40)	0.95 (0.78–1.17)		
Single, no children	0.90 (0.68–1.18)	1.12 (0.90–1.40)	0.90 (0.74–1.09)		
Single, with children	0.96 (0.64–1.45)	1.28 (0.94–1.74)	1.00 (0.76–1.33)		
***Country of birth***				0.139	0.048
Sweden	Ref	Ref	Ref		
Elsewhere	**1.24 (1.01–1.53)**	1.12 (0.96–1.30)	1.02 (0.88–1.17)		
***Place of residence***				**0.022**	0.085
Big cities	Ref	Ref	Ref		
Intermediate/small cities	1.47 (0.88–2.47)	**1.69 (1.19–2.39)**	1.32 (0.94–1.85)		
***Year of education***				**< 0.001**	0.388
0–9 years	Ref	Ref	Ref		
10–12 years	0.79 (0.60–1.03)	0.94 (0.75–1.17)	0.98 (0.80–1.20)		
> 12 years	**0.50 (0.37–0.67)**	**0.68 (0.54–0.86)**	**0.78 (0.63–0.97)**		
***Unemployment***				0.427	0.024
none	Ref	Ref	Ref		
any	1.24 (0.91–1.68)	0.92 (0.70–1.21)	1.08 (0.86–1.37)		
***Profession***				**< 0.001**	1.249
Occupations with university requirements	Ref	Ref	Ref		
Administrative roles and customer service	**1.37 (1.05–1.79)**	1.00 (0.84–1.20)	**1.30 (1.11–1.54)**		
Non-managerial workers	**1.72 (1.25–2.38)**	0.97 (0.77–1.24)	**1.50 (1.21–1.84)**		
Not gainfully employed	1.16 (0.83–1.61)	**0.36 (0.26–0.49)**	**0.43 (0.32–0.57)**		
No information on occupation	**1.43 (1.04–1.97)**	0.86 (0.68–1.09)	1.00 (0.81–1.25)		
**Clinical-related factors**					
***Type of diagnostic category*** ^***3***^				**< 0.001**	4.783
Anxiety disorders	Ref	Ref	Ref		
Mood/affective disorders	**1.48 (1.06–2.07)**	**1.50 (1.16–1.95)**	**1.30 (1.04–1.63)**		
Stress-related disorders	**2.29 (1.71–3.06)**	**2.99 (2.43–3.67)**	**2.00 (1.67–2.39)**		
Anxiety- and mood/affective disorders	**1.66 (1.12–2.47)**	1.33 (0.94–1.90)	**1.72 (1.30–2.26)**		
Anxiety- and stress-related disorders	**2.83 (2.00–4.01)**	**4.19 (3.28–5.36)**	**3.25 (2.62–4.04)**		
Mood/affective and stress-related disorders	**5.36 (3.74–7.68)**	**6.88 (5.25–9.02)**	**3.82 (2.94–4.95)**		
Anxiety- mood/affective- and stress-related disorders,	**5.36 (3.55–8.11)**	**7.72 (5.59–10.7)**	**4.98 (3.65–6.80)**		
***Comorbid psychological disorders in primary care***				0.016	0.091
None	Ref	Ref	Ref		
Any	0.97 (0.68–1.40)	**0.55 (0.38–0.82)**	0.95 (0.70–1.29)		
***(Post) Psychiatric diagnosis from inpatient/specialised outpatient care***				**< 0.001**	4.047
None	Ref	Ref	Ref		
Any	**6.79 (5.49–8.40)**	**3.68 (3.12–4.33)**	**2.14 (1.82–2.53)**		
***(Prior) Somatic treatment in inpatient/specialised outpatient care***				**< 0.001**	0.580
None	Ref	Ref	Ref		
Any	**1.92 (1.53–2.40)**	**1.20 (1.04–1.39)**	**1.55 (1.35–1.79)**		
***(Post) Somatic treatment in inpatient/specialised outpatient care***				**< 0.001**	0.419
None	Ref	Ref	Ref		
Any	**1.51 (1.21–1.88)**	**1.23 (1.06–1.42)**	**1.54 (1.34–1.77)**		
***Systematic psychological treatment*** ^***4***^				0.066	0.141
None	Ref	Ref	Ref		
In year one	**1.38 (1.09–1.75)**	0.97 (0.81–1.16)	1.01 (0.85–1.18)		
In years two - three (only)	1.14 (0.80–1.61)	0.96 (0.74–1.25)	1.21 (0.97–1.52)		
In years one and two to three	**1.63 (1.16–2.30)**	1.08 (0.82–1.42)	1.03 (0.80–1.35)		
***Purchase of psychotropics*** ^***5***^				**< 0.001**	0.987
None	Ref	Ref	Ref		
In year one	1.18 (0.90–1.55)	**1.66 (1.40–1.96)**	**1.25 (1.07–1.47)**		
In year two - three (only)	**1.56 (1.08–2.25)**	**1.36 (1.03–1.79)**	**1.56 (1.23–1.97)**		
In years one to three	**2.34 (1.83–2.98)**	**1.83 (1.53–2.19)**	**1.51 (1.28–1.78)**		
***Purchase of any other psychotropics*** ^***6***^				**0.044**	0.071
None	Ref	Ref	Ref		
Any	1.32 (0.98–1.79)	0.77 (0.56–1.05)	0.96 (0.72–1.28)		

^1^ Difference in Nagelkerke R^2^ between the fully adjusted and the reduced models, excluding one covariate at a time

^2^ These factors were measured on the 31st of December 2016

^3^ Anxiety disorders (International Statistical Classifications of Diseases – version 10 (ICD10): F40-F42); Mood/affective disorders (ICD10: F32-F39); Stress-related disorders (ICD10: F43)

^4^ Systematic psychological treatment included psychodynamic treatment (PDT), cognitive psychological therapy (CT), cognitive behavioural therapy (CBT), mentalization-based therapy (MBT), Eye Movement Desensitization and Reprocessing (EMDR), systemic therapy (ST), dialectical-behavioural therapy (DBT), interpersonal therapy (IPT), and other psychological treatment (KVÅ codes: DU008- DU011, DU013-DU014, DU020-DU022)

^5^ Psychotropic medications included antidepressants (Anatomical Therapeutic Chemical Classification (ATC): N06A), anxiolytics (ATC: N05B), hypnotics and sedatives (ATC: N05C) and alimemazin (R06AD01)

^6^ ATC: N05A, N03AF01,N03AG01,N03AX09,N05AN01,N06B, N07BB, N07BC, N06CA

Additional crude multinomial logistic regressions were performed for all covariates. In this crude analysis, a larger number of significant associations were found between treatment variables and work disability; however, after adjusting for the other covariates, these effects decreased, indicating that a portion of the effects are accounted for in the variation of the different variables.

### Sociodemographic and work-related factors

Differences in trajectories of work disability were found between professions. The fully adjusted odds ratios (ORs) reveal that non-managerial workers (OR: 1.72; 95% CI: 1.25–2.38) and workers within the administration and customer services (OR: 1.37; 95% CI:1.05–1.79) had a higher probability of belonging to the *high-increasing* trajectory as well as to the *low-increasing* trajectory (OR: 1.50; 95% CI: 1.11–1.54 and OR: 1.30; 95% CI: 1.21–1.84, respectively) than individuals in occupations with university requirements. With regards to age, individuals in the age group 60–62 years had a considerably higher probability of belonging to the *high-increasing* trajectory with high levels of work disability (OR: 6.28; 95% CI: 4.08–9.68), but also to the *low-increasing* trajectory of work disability (OR: 2.21; 95% CI: 1.63–3.01) compared to the youngest age group of 22–29 years. Generally, higher ages were associated with an increasing probability of belonging to the *high-increasing* trajectory of work disability (OR range 1.68–6.28).

### Clinical-related factors

The type of diagnostic category explained the most significant variance in trajectory group membership according to the R^2^ value. Compared to individuals with anxiety disorders alone, those with stress-related disorders, either in isolation (OR: 2.29; 95% CI: 1.71–3.06) or in combination with anxiety disorders (OR: 2.83; 95% CI: 2.00–4.01) or mood/affective disorders (OR: 5.36; 95% CI: 3.74–7.68), had the highest probability of being in the *high-increasing* trajectory of work disability. A similar trend was also seen for belonging to the *low-increasing* trajectory group, albeit with lower odds ratios. Receiving a diagnosis for a psychiatric disorder in inpatient/specialised outpatient healthcare within three years following the new episode of any anxiety-, mood/affective-, or stress-related disorder was strongly associated with an increased probability of belonging to the *high increasing* (OR 6.79; 95% CI: 5.49–8.40), *peak* (OR 3.68; 95% CI: 3.12–4.33) as well as the *low increasing* (OR 2.14; 95% CI: 1.82–2.53) trajectories.

Purchases of psychotropic medication after the diagnosis of any anxiety-, mood/affective-, or stress-related disorder were associated with higher odds of following a *high-increasing* (OR: 2.34; 95% CI: 1.83–2.98), *peak* (OR: 1.83; 95% CI: 1.53–2.19) or *low-increasing* (OR: 1.51; 95% CI: 1.28–1.78) trajectory of work disability, compared to the c*onstant low* group. Furthermore, there were tendencies for individuals who received psychological therapy in the year following the episode of the anxiety-, mood/affective-, or stress-related disorder to be more likely to belong to the *high-increasing* group than the *constant low* group, as compared to those who did not receive psychological therapy (OR: 1.38; 95% CI: 1.09–1.75). Individuals who received systematic psychological treatment in all three years of the follow-up period had even higher odds of following the *high-increasing* trajectory of work disability (OR: 1.63; 95% CI: 1.16–2.30), compared to the *constant low* group in contrast to individuals who did not receive therapy.

## Discussion

In this sizeable register study of individuals experiencing a new episode of anxiety-, mood/affective-, or stress-related disorders treated in primary healthcare settings, we identified four distinct trajectory groups: *high-increasing* (5.1% of the population), characterised by high levels of work disability during follow-up, *peak* (11.1%), with a peak around the time of diagnosis, *low-increasing* (12.8%), with increases in work disability throughout the study period and *constant-low* (71.1%), with low levels of average work disability over the study period. Stress-related disorders, either alone or in combination with mood/affective disorders or anxiety disorders, a diagnosis of any psychiatric disorder in secondary healthcare following the diagnosis of an anxiety-, mood/affective-, or stress-related disorder, purchases of psychotropic medication, having a non-managerial, administrative, or customer service occupation, and higher age at diagnosis were most strongly associated with trajectory group membership.

### Trajectory groups

Nearly three-quarters of all participants in this study experienced a *constant low* trajectory of work disability, with none or very little work disability, during the three-year follow-up period. In a previous study by our group, we examined trajectories of work disability in a population of individuals treated for CMDs in secondary healthcare (who can be assumed to have a higher level of illness severity than the current population) [5]; in comparison, individuals in the present study had a markedly better connection to the labour market. Consistent with our prior work, we observed that individuals who were previously treated for psychiatric disorders in secondary care had a much higher risk of following a high work disability trajectory. Those belonging to the trajectory group with *low increasing* work disability had a relatively modest increasing trend of work disability, with about 20 days of WD in the last six-month interval. Whilst long-term work disability is considered a poor outcome, periods of short-term sickness absence may be beneficial for maintaining a sustainable work-life [21]. The curve of days of work disability for those following the *low-increasing* trajectory did, however, increase during the follow-up, which might lead to high levels of work disability some years after the diagnosis. Here, measures might be taken early to prevent long future spells of work disability.

The *increasing-high* trajectory group, who experienced a rapidly increasing pattern of work disability some years before the diagnosis of the anxiety-, mood/affective-, or stress-related disorder, continued to grow in the years after the diagnosis and accounted for an average of 80 days with work disability three years after the diagnosis. From a welfare perspective, this group is arguably the most challenging as such individuals are at risk of being dependent on welfare benefits over the long term. Reducing days of work disability within this group has the potential to both decrease welfare costs for societies and increase well-being among individuals, thereby enhancing a sustainable working life.

The *Peak* trajectory of work disability, indicating a recovery in the time after the diagnosis, is, from a welfare perspective, the most optimal group. Those following this trajectory have an increase in work disability around the time of diagnosis but have a relatively rapid recovery and have a much-reduced work disability two years after the diagnosis. This group might, therefore, not require extra support to reduce days on work disability.

### Predictors of trajectory groups

#### Sociodemographic and work-related factors

We found that profession was an important factor in explaining the differences between trajectory groups of work disability among individuals diagnosed with anxiety-, mood/affective-, or stress-related disorders in primary health care. Non-managerial workers had a high probability of belonging both to the *high-increasing* trajectory and the *low-increasing* trajectory of work disability compared to individuals with occupations with university requirements. Earlier studies describe this and have suggested that such findings may be explained by less flexible working patterns, meaning that individuals cannot decide when or where to complete their work activities [22]. Also, those working within the administration and customer support had a high probability of belonging to either of the increasing trajectories of work disability. Therefore, these groups might be prioritised by the stakeholders within rehabilitation to reduce the risk of having long periods of work disability.

In general, age was considered an essential determinant of work disability. Therefore, our finding that age was among the strongest predictors of trajectory group membership is not unexpected. Nevertheless, the exceptionally high probability of sickness absence among those aged 60–62 is a novel finding. The retirement age has risen worldwide, including in Sweden [23], and is expected to increase further in parallel with global trends in life expectancy. Policies, not least Agenda 2030, stipulate a sustainable work-life throughout life [24]. Whilst these findings require further replication, they suggest that enabling persons over 60 to work with anxiety-, mood/affective-, or stress-related disorders is crucial to reaching sustainable work among individuals near retirement.

#### Clinical-related factors

Those with stress-related disorders, either alone or in combination with mood/affective disorders and anxiety disorders, were associated with the highest probability of belonging to the *high increasing* trajectory. Such findings are important given that studies have shown that stress-related disorders appear to be increasing [25]. One must, however, keep in mind that other reactions to severe stress (ICD-10: F48.8) in Sweden allow for much more extended periods of sickness absence compared to depressive disorders or anxiety disorders, so the number of individuals diagnosed with exhaustion disorder might, therefore, be a consequence of the administrative regulations within the Social insurance scheme rather than a marker of severity of the disorder [26]. Job demands have increased much during the past decades, and if high job control and support do not mitigate the effect of high job demands, the health consequences can lead to work disability [7, 27–29]. Also, the length of the sickness period at baseline seems to affect the ability to have a sustainable working life in the future. In a previous study from our research group, we found that individuals diagnosed with stress-related disorders in isolation were less likely to receive psychological treatment and psychotropic medication [30] and, as a consequence, may experience a higher degree of work disability.

With regards to clinical-related factors, we observed that individuals who had purchased psychotropic medications had a higher probability of belonging to the *high-increasing* work disability group. Such findings are likely to reflect confounding by indication, whereby individuals who are the most unwell (and therefore most likely to receive work disability payments) are the most likely to receive medication and systematic psychological treatment. We found, most probably due to confounding by indication, only limited evidence to suggest that receipt of neither a systematic psychological treatment nor medication was associated with the trajectory groups of work disability. These findings are, to some extent, also consistent with previous studies [10, 11]. Using the current study design, we cannot determine whether the most unwell individuals experienced an improvement in work disability following pharmacological treatment. An implication of this is that studies using statistical methods to account for treatment allocations in real-world practice are needed to fully assess the effect of confounding by indication and thereby estimate the impact of treatment on work disability.

### Strengths and limitations

The strengths of this study include the use of high-quality register data, including individual-level information on several sociodemographic, work and clinical-related covariates [31, 32]. Also, the population-based design minimised the follow-up loss, including all adults between 22 and 62 who lived in Stockholm County and were diagnosed with anxiety-, mood/affective-, or stress-related disorders, and the study period of six years enabled us to assess the development of sickness absence both before and after a diagnosis.

There are also limitations worth mentioning. Data on sickness absence only includes information on spells longer than 14 days, as the employers cover the first two weeks and do not register these days. This might slightly underestimate the risk of work disability among those with anxiety-, mood/affective-, or stress-related disorders. Given the study design, this is not a significant problem as we are interested in more prolonged sickness absence spells. Moreover, we only know about receiving psychological therapy in primary care. Although VAL includes many private clinics, we may lose some individuals treated in private clinics. As our study included only individuals residing in Region Stockholm, a highly affluent region in Sweden, our findings may not be generalised to individuals living in rural parts of a country. Due to the risk of confounding by indication, our findings regarding associations between treatment receipt and work disability must be interpreted cautiously. Another crucial limitation regarding the validity of results pertains to the lumping together of very different types of disorders under rubrics, such as “stress-related disorders” (including both post-traumatic disorder and adjustment disorder) and “anxiety disorders” (including a broad range of very different disorders such as obsessive-compulsive disorder (OCD) and panic disorder). According to the Swedish National Board of Health and Welfare, these disorders have significantly differential recommendations for prescribing sick leave [26]. Our study findings cannot be generalised to all people with anxiety-, mood/affective-, or stress-related disorders, as our sample is based on individuals who seek healthcare and who received a diagnosis in primary care. In Europe, for example, refugees have been reported to have limited access to mental healthcare compared to their native-born peers [33]. In a review, about 50–60% of those who fulfil the criteria for being diagnosed with a CMD did not get any professional help or treatment [34]. Our analysis strategy is designed to capture changes in the outcome variable over time. For this, a meaningful event, in this case, a diagnosis of anxiety-, mood/affective-, or stress-related disorders, is required to measure changes occurring before and after this date (T0 in our analysis). Had we not implemented this restriction, individuals entering the cohort could be at markedly different stages of illness, which would likely influence their level of work disability. As we had seven different combinations of subgroups of anxiety-, mood/affective-, or stress-related disorders, we could not examine separate trajectories for all these groups. Moreover, sex was not strongly associated with membership in any trajectory groups in our study; we therefore chose not to stratify on sex. Assessing sex differences within treatment for anxiety-, mood/affective-, or stress-related disorders would, however, be the scope of future research. Finally, the results should be interpreted in light of the fact that the exposures were measured at the time of the diagnosis, which means that the outcome partly preceded the exposures.

## Conclusions

Whilst the majority of individuals treated for anxiety-, mood/affective-, or stress-related disorders in primary care seem to have a good prognosis concerning work disability, many individuals do experience increasing levels of work disability in the three years that follow. Those having a diagnosis of stress-related disorders, being aged 60 years and above, receiving psychotropic medication, having a diagnosis of a psychiatric disorder from an inpatient and specialised outpatient healthcare, and having a non-managerial occupation or being employed within the administration and customer service were associated with a higher likelihood of extended work disability. As such, these individuals could benefit from additional support early in the disease spell.

## Electronic supplementary material

Below is the link to the electronic supplementary material.

Supplementary Material 1

## Data Availability

The data used in this study cannot be publicly available due to privacy regulations. According to the General Data Protection Regulation, the Swedish law SFS 2018:218, the Swedish Data Protection Act, the Swedish Ethical Review Act and the Public Access to Treatment of common mental disorders in primary care. Readers may contact Kristina Alexanderson (kristina.alexanderson@ki.se) regarding the data.
