# Supplementary material for: Trajectories of work disability among individuals with anxiety-, mood/affective-, or stress-related disorders in a primary healthcare setting
Source: BMC Psychiatry. 2024 Sep 19;24:623. doi: 10.1186/s12888-024-06068-5 (PMC11414192; doi:10.1186/s12888-024-06068-5)
Supplement: Supplementary file 1 — Supplementary Material 1 [file 12888_2024_6068_MOESM1_ESM.docx]

**Supplementary Table 1.** Swedish register data,sources and utilisation.

| **Register** | **Source** | **Utilisation** |
| --- | --- | --- |
| VAL, *the data storage of healthcare data from Region Stockholm, Sweden.* | Region Stockholm | Variables related to treatment and diagnoses in primary care. |
| National Patient Registers (NPR) | National Board of Health and Welfare | Variables related to treatment in inpatient and specialised outpatient care. |
| Prescribed Drug Register (PDR) | National Board of Health and Welfare | Variables related to medication purchases. |
| Longitudinal Integration Database for Health Insurance and Labour Market Studies (LISA) | Statistics Sweden | Sociodemographic, residency and employment variables |
| Micro-Data for Analyses of Social Insurance (MiDAS) | Social Insurance Agency | Variables related to work disability (sickness absence and disability pension) |
